# Microstructure and Electrical Properties of AZO/Graphene Nanosheets Fabricated by Spark Plasma Sintering

**DOI:** 10.3390/ma9080638

**Published:** 2016-07-29

**Authors:** Shuang Yang, Fei Chen, Qiang Shen, Enrique J. Lavernia, Lianmeng Zhang

**Affiliations:** 1 State Key Laboratory of Advanced Technology for Materials Synthesis and Processing, Wuhan University of Technology, Wuhan 430070, China; shuang_yang@yeah.net (S.Y.); sqqf@263.net (Q.S.); lmzhang195501@126.com (L.Z.); 2 Department of Chemical Engineering and Materials Science, University of California, Davis, CA 95616, USA; lavernia@ucdavis.edu

**Keywords:** graphene nanosheets, Al-doped-ZnO, electrical properties, spark plasma sintering

## Abstract

In this study we report on the sintering behavior, microstructure and electrical properties of Al-doped ZnO ceramics containing 0–0.2 wt. % graphene sheets (AZO-GNSs) and processed using spark plasma sintering (SPS). Our results show that the addition of <0.25 wt. % GNSs enhances both the relative density and the electrical resistivity of AZO ceramics. In terms of the microstructure, the GNSs are distributed at grain boundaries. In addition, the GNSs are also present between ZnO and secondary phases (e.g., ZnAl_2_O_4_) and likely contribute to the measured enhancement of Hall mobility (up to 105.1 cm^2^·V^−1^·s^−1^) in these AZO ceramics. The minimum resistivity of the AZO-GNS composite ceramics is 3.1 × 10^−4^ Ω·cm which compares favorably to the value of AZO ceramics which typically have a resistivity of 1.7 × 10^−3^ Ω·cm.

## 1. Introduction

The Li-ion batteries using liquid or organic electrolytes have been widely investigated for the application in electric vehicles(EV) and backup uninterruptible power supply (UPS) systems. Challenges in terms of scale-up and guaranteed safety have been proposed by many researchers. Therefore, all-solid-state batteries (ASSB) have been designed which show significant improvements in energy density and safety relative to those of commonly used organic liquid and polymer-based lithium-ion batteries. In a related work, Baek et al. [1] fabricated an all-solid-state lithium rechargeable battery with both the electrode and electrolyte processed by spark plasma sintering (SPS). The battery is characterized by a carefully designed architecture with a good lithium diffusion percolation path and low solid contact resistance without any defects and undesirable reactions arising from the sintering method. The more widely used solid-state synthesis of ABBS attaches much importance to synthesizing cathode materials.

As a result of the excellent electrical and optical properties [2], transition metal oxide–based materials have found widespread applications in electrode active materials, such as Ni-MH cells, lithium cells, solar cells, and fuel cells, due to their high electrochemical activity [3]. Recently, transition metal oxides have evolved as potential electrode materials for lithium-based batteries due to their higher theoretical capacity and non-reactive properties relative to those of conventional carbon-based materials. Among the transition metal oxides, ZnO possesses numerous advantages, such as low cost, manufacturability and environmental inertness [4]. As such, zinc oxide is a promising substitute for conventional graphite anodes in lithium-ion batteries due to its superior theoretical capacity (978 mAh·g^−1^) which compares favorably to the theoretical capacity of graphite (372 mAh·g^−1^) [5].

ZnO is a well-known wide band gap (3.37 eV) semiconductor with a large binding energy (60 meV) and a proven capacity for reversible electrochemical Li storage [6,7]. Feng et al. [8] reported that ZnO nanoplates used as anodes for Li-ion batteries exhibited a very high capacity of ~680 mAh·g^−1^, with continuous fading for a stable capacity of ~180 mAh·g^−1^. Recently, Bresser et al. [9] utilized ZnO nanoparticles as anode materials for lithium-ion batteries and reported a reversible capacity of ~700 mAh·g^−1^ in the first cycle, but it rapidly faded during subsequent charge-discharge cycles before stabilizing at ~260 mAh·g^−1^. Hence, on the basis of these published results, it is evident that ZnO is an attractive candidate material for anodes because of its inherent properties, which include low reversible capacity and a severe capacity fading response. There are, however, important technical barriers that need to be addressed before ZnO can be successfully used as anodes, such as a low electrical conductivity, which hinders the practical application of ZnO-based anodes in lithium-ion batteries [10].

When used as anodes for Li-ion batteries, graphene-based metal oxides display significantly improved performance in terms of cycling stability and rate capability [11,12,13,14]. Furthermore, published results suggest that graphene nanosheets may enhance the electronic conductivity of the overall electrode, as well as stabilize the microstructure of metal oxides during cycles [15,16,17]. These effective methods have also shown much impact on the improvement of the electrochemical performance for ZnO. In one study, metal-doped ZnO nanoparticles and carbon-based composites (e.g., carbon-coated ZnO nanorods) [18,19] were prepared for use as anodes in lithium-ion batteries. Moreover, other studies have shown that the electrochemical performance is significantly improved by the substitution of zinc by iron within the wurtzite lattice with a reversible capacity of ~410 mAh·g^−1^ after 100 cycles compared to pure zinc oxide of ~260 mAh·g^−1^ [9]. In addition, in a related study, a Li-ion cell assembly was prepared consisting of a ZnO multiwalled carbon nanotube nanocomposite free-standing anode and a Li metal cathode, and the results reveal an excellent discharge capacity, remaining as high as 460 mAh·g^−1^ after 100 cycles [20]. Thus, it is evident that ZnO-based ceramics should be investigated as promising anode materials for all-solid-state lithium rechargeable batteries.

In view of the above discussion, the present work was motivated by the following three questions. First, what is the feasibility of using spark plasma sintering (SPS) to fabricate AZO-GNSs composite ceramics? Second, what are the corresponding densification behavior, microstructure characteristics and electrical properties? Third, what are the underlying mechanisms that govern the electrical response and what is the influence, if any, of the spatial distribution of GNS?

## 2. Experimental Procedures

Graphene nanosheets produced using a modified Hummer’s method, were purchased from NANO XF (Nanjing, China). The resultant platelets were approximately 0.8–1.2 nm in thickness and 0.5–2 mm in length. As-received AZO nanoparticles (Huzheng Nanotechnology Co. Ltd., Shanghai, China) were used in this study. The composition of the AZO nanoparticles is Al_2_O_3_/ZnO of 2/98 (mol %); purity > 99.9%; specific surface area of 30–50 m^2^/g; apparent density of 1.21 g/cm^3^; average particle size of 80~100 nm; and resistivity of 1.2 × 10^−1^ Ω·m.

The as-received GNSs were dispersed in a solution of dimethyl formamide (DMF) with ultrasonic agitation for 1 h. Then, AZO powders were added to these slurries at GNSs ratios of 0, 0.025, 0.05, 0.1 and 0.2 wt. %, and further sonicated for 30 min. The compounds were then milled at 300 rpm by a planetary ball mill (QM-3SP2, Nanjing, China) for 24 h with a weight ratio of ball to powder of 3:2, and DMF as a solvent. The composite powders were then dried at 100 °C using a vacuum drying oven for 24 h. The composite powders were loaded into a 20-mm-diameter graphite die inside the SPS furnace (SPS-1050, Tokyo, Japan). A piece of graphitic paper was placed between the punch and powder in order to facilitate removal of the sintered sample. Subsequently, the composite powders were sintered at 900 °C, 1000 °C, 1100 °C and 1200 °C from room temperature with a temperature rate of 100 °C/min. The applied uniaxial pressure was 40 MPa and dwell time was 3 min under a vacuum of 20 Pa. The temperature was monitored using an infrared instrument during the sintering process. The obtained bulk with the GNSs content of 0, 0.025, 0.05, 0.1 and 0.2 are hereby designated as AZO, AZO-0.025G, AZO-0.05G, AZO-0.1G and AZO-0.2G, respectively.

The density of AZO-GNSs composite ceramics were measured via Archimedes’ method using deionized water immersion, and the relative density was calculated by taking the theoretical density of AZO as 5.67 g/cm^3^. The carrier concentration, Hall mobility and resistivity of AZO ceramics were measured at room temperature using the Hall measurement system (Accent, HL5500PC, Hertfordshire, UK) according to the van der Pauw method [21]. The microstructures of the sintered ceramics were observed by field emission scanning electron microscopy (FESEM, 20 kV, FEI Quanta FEG250, Hillsboro, OR, USA) and a high-resolution transmission electron microscopy (JEM-2100F STEM, Tokyo, Japan), The average grain size was measured by the intercept-line method, using 1.225 as the stereological correction factor. Approximately 200 grains were used for each data set reported [22].

## 3. Results

### 3.1. Phase Composition and Microstructure of AZO-G Composite Nanoparticles

**Figure 1:**
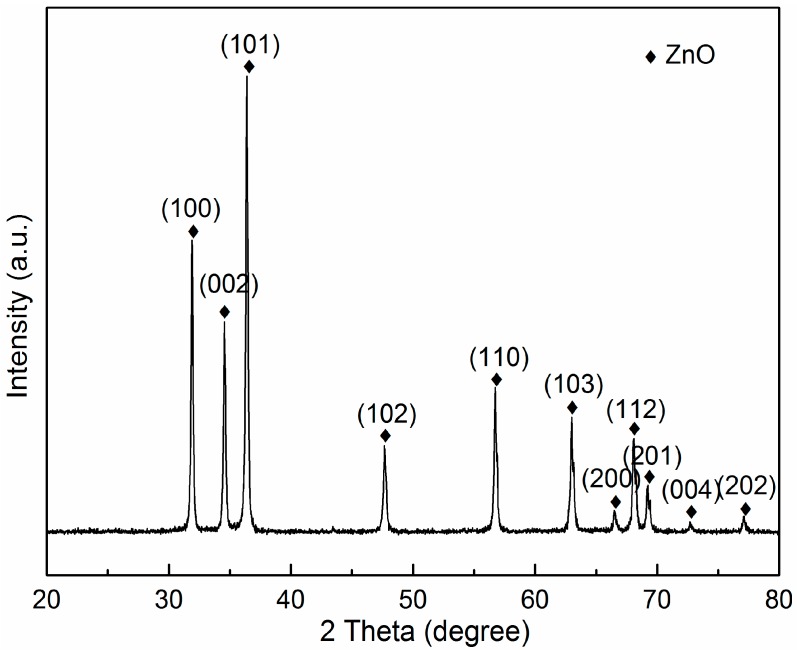
XRD patterns of AZO-0.05G composite powder.

**Figure 2:**
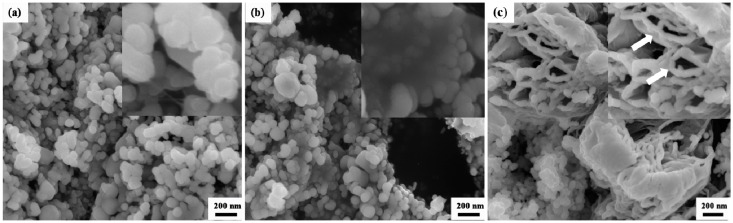
SEM images of composite powders: (**a**) AZO-0.025G; (**b**) AZO-0.05G; (**c**) AZO-0.1G.

Figure 1a shows the XRD patterns for AZO-0.05G composites nanoparticles following ball milling for 24 h. It can be observed that all diffraction peaks are consistent with those of ZnO (PDF #36-1451), with a quartzite structure indicating only one crystalline phase related to ZnO. The peaks of GNSs in XRD patterns are not observed due to the ultra-low percentage of GNSs in the as-prepared composite powders. A macroscopic view of the AZO nanoparticles after ball milling with 0.025 wt. % GNSs is evident in Figure 2a. The grain size of AZO-0.025G composite nanoparticles is approximately 100 nm. The thin transparent GNSs are not observed in AZO-0.025G composite powders and there are no obvious agglomerations in the composite powder after ultrasonication and ball milling. The microstructure of AZO-0.05G and AZO-0.1G composite powders is shown in Figure 2b,c. In the case of the AZO-0.05G composite powders, the transparency indicates that the thin platelets contained only a few graphene nanosheets. The graphene nanosheets are well distributed throughout the AZO-0.05G powder and reveal no agglomeration. However, in the case of a high volume of GNSs, as in AZO-0.1G, agglomerates of graphene nanosheets were observed in some regions. An example of large agglomerates of segregated GNSs in AZO-0.1G composite powder is shown in Figure 2c by the arrow.

### 3.2. Relative Density and Grain Size of Composite Ceramics

Table 1 shows the relative density and grain size of the AZO-G composite ceramics for different sintering temperatures and with different GNSs contents. The relative density of pure AZO ceramic increases as the sintering temperature varies from 1000 to 1200 °C and it attains a maximum value at the sintering temperature of 1200 °C. However, in the case of the AZO-0.025G composite ceramics, the relative density increases from 98.3% to 99.2% when the sintering temperature increases from 1000 to 1100 °C. Increasing the temperature to 1200 °C, however, leads to a decrease in the relative density of the AZO-0.025G composite ceramics. Furthermore, the AZO-G composite ceramics with 0.025 wt. % GNSs attain the highest relative density at the sintering temperature of 1100 °C. It can be seen from Table 1 that the relative density of AZO-G composite ceramics decreases slightly with the increase of GNSs, and this is attributed to the agglomeration of the graphene nanosheets.

The AZO-0.025G composite ceramic exhibits limited grain refinement, as shown in Table 1. The average grain size of AZO-0.025G composite ceramics sintered at 1100 °C is 2.8 μm. A well-known response of GNSs during sintering is a phenomenon described as grain wrapping [23], where GNSs act as diffusion barriers and hence effectively pin the grains by wrapping themselves around them. In the case of lower volume fractions of GNSs (e.g., AZO-0.025G), only a few grains are wrapped and pinned. Although partial grain pinning may lead to a decrease in the grain size, it also promotes abnormal grain growth in AZO. This phenomenon, which leads to grain refinement in some regions and abnormal grain growth in others, has been reported in other studies [24]. From Reference [24], it is clear that Al_2_O_3_/graphene composites show no significant grain refinement with the graphene volume fraction of 5%. However, with the increase of the volume fraction of graphene from 5% to 15%, the grain size for Al_2_O_3_/graphene has been significantly decreased. As another example, TaC/graphene composites [25] exhibit a limited grain refinement with the addition of graphene (1 vol. %) and a sharply decreased grain size of TaC/graphene composites with the addition of graphene (3 and 5 vol. %). Similarly, the extent of grain refinement is significant when the volume fraction of GNSs is increased in our work. The average grain size of AZO-0.05G and AZO-0.1G composite ceramics is 1.5 and 0.9 μm, respectively. In this case, a high volume fraction of GNSs will effectively stabilize the grains as a higher percentage of them are “wrapped”.

### 3.3. Microstructure of AZO-G Composite Ceramics

**Figure 3:**
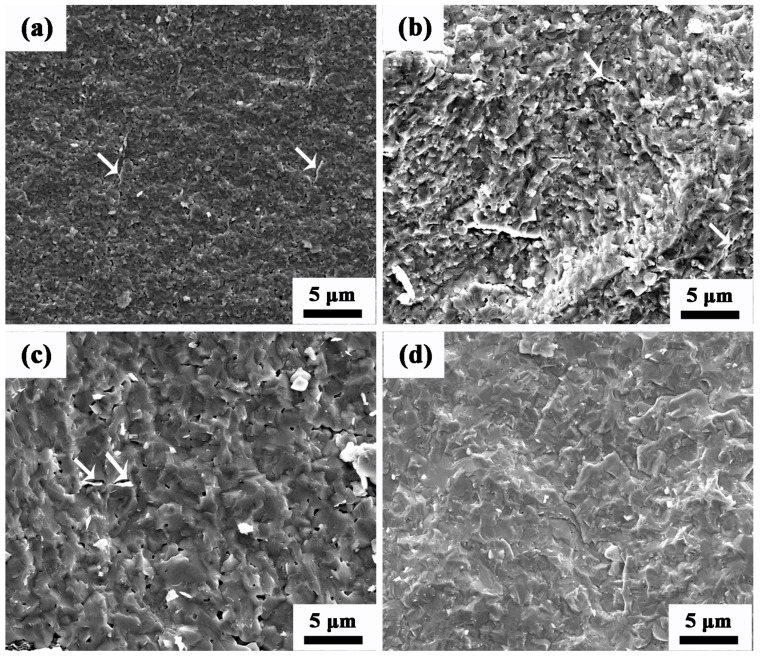
SEM micrographs of AZO-0.05G composite ceramics at sintering temperatures of: (**a**) 1000 °C; (**b**) 1100 °C; (**c**) 1200 °C; (**d**) 1300 °C.

The SEM micrographs of the fracture surface of the AZO-0.05G composite ceramics sintered at various temperatures are shown in Figure 3. As seen in the figure, the distribution of GNSs is homogenous within the AZO ceramic matrix for the range of sintering temperatures studied, 1000 °C, 1100 °C and 1200 °C. It is clear that the microstructure of AZO-0.05G is dense following sintering at 1000 °C. There is, however, some distributed porosity that can be seen on the fracture surfaces corresponding to a sintering temperature of 1200 °C, indicating a low relative density for the AZO-0.05G composite ceramics.

**Figure 4:**
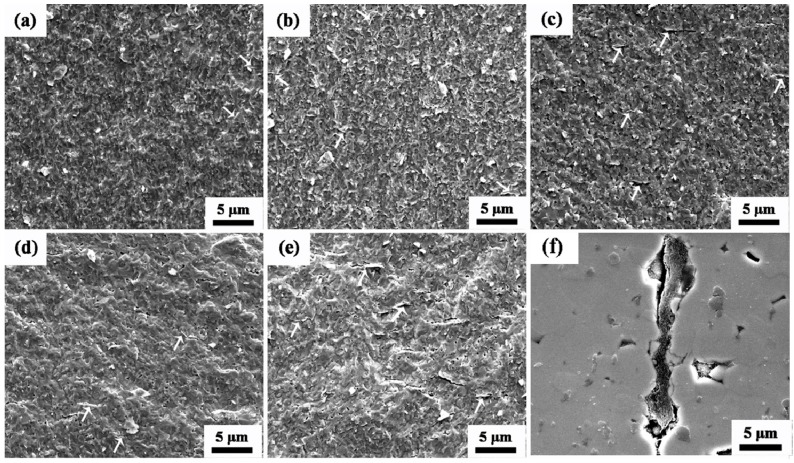
SEM micrographs of AZO-G ceramics with GNS content of (**a**) 0 wt. %; (**b**) 0.025 wt. %; (**c**) 0.05 wt. %; (**d**) 0.1wt. %; (**e**) 0.2 wt. % and (**f**) 0.2 wt. % (polished surface).

Figure 4 shows the fracture surface corresponding to the AZO ceramic and AZO-G composite ceramics with various GNS contents, respectively. The results show that uniformly distributed GNSs in the AZO matrix (highlighted by white arrows) have no apparent influence on the relative density of AZO-G composite ceramics as the GNS content varies from 0.025 to 0.05 wt. %. For AZO-0.1G and AZO-0.2G, however, significant cracks around graphene nanosheets are observed in Figure 4f due to the agglomeration of GNSs, ultimately affecting the relative density of the AZO-G composite ceramics shown in Table 1.

**Figure 5:**
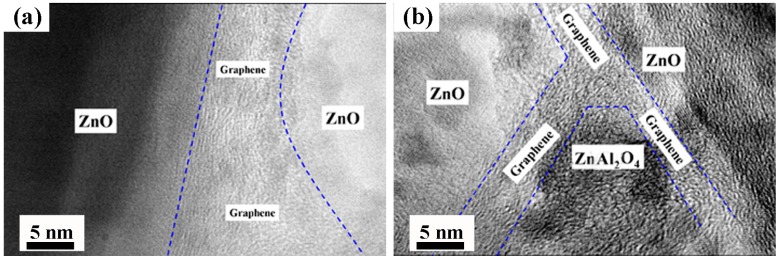
HRTEM image of AZO-0.05G composite ceramics: (**a**) GNSs at ZnO/ZnO grain boundary; (**b**) GNSs at ZnO/ZnAl_2_O_4_ grain boundary and grain boundary triple junction.

Figure 5 shows HRTEM images of the spatial distribution of GNSs in the AZO matrix. As seen from Figure 5a, GNSs with a thickness of about 10 nm are located between ZnO/ZnO grain boundaries and the interface between ZnO and graphene appears to be free from other impurities. Another unique and significant characteristic in the microstructure of AZO-G composite ceramics shown in Figure 5b is that, although GNSs are generally flat and straight at ZnO/ZnO grain boundaries, they are also observed at a ZnO/ZnAl_2_O_4_ grain boundary and are connected to each other to form a network at a grain boundary triple junction.

### 3.4. Electrical Properties of AZO-G Composite Ceramics

**Figure 6:**
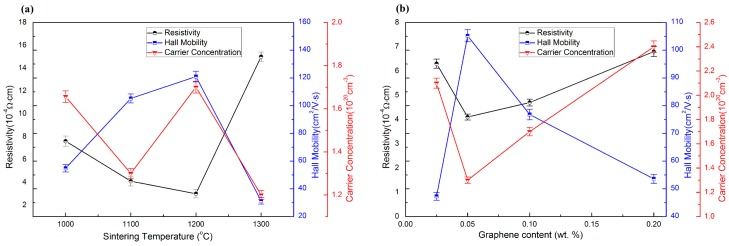
The electrical property of AZO-G composite ceramics: (**a**) at various sintering temperature; (**b**) with various graphene nanosheets content.

Figure 6a shows the room temperature resistivity, carrier concentration and Hall mobility for the AZO-0.05G composite ceramics as a function of the sintering temperature. The results show a decrease of resistivity for AZO-0.05G composite ceramics as the sintering temperature is increased from 1000 to 1200 °C. However, the resistivity of AZO-0.05G composite ceramics exhibits a sharp increase at the sintering temperature of 1300 °C. The deterioration of resistivity is attributed to the oxidation of GNSs in AZO-0.05G, which is apparent in Figure 4d. It is clear that the resistivity of AZO-0.05G composite ceramics sintered at 1100 °C attains a minimum value of 4.1 × 10^−4^ Ω·cm, as shown in Figure 6b. As the content of GNSs ranges from 0.05 to 0.2 wt. %, the resistivity and Hall mobility reveal a increase and sharp decrease, respectively, which can be explained on the basis of a deterioration in relative density caused by the agglomeration of graphene nanosheets.

## 4. Discussion

### 4.1. Influence of GNSs on Densification of AZO-GNSs during SPS

**Figure 7:**
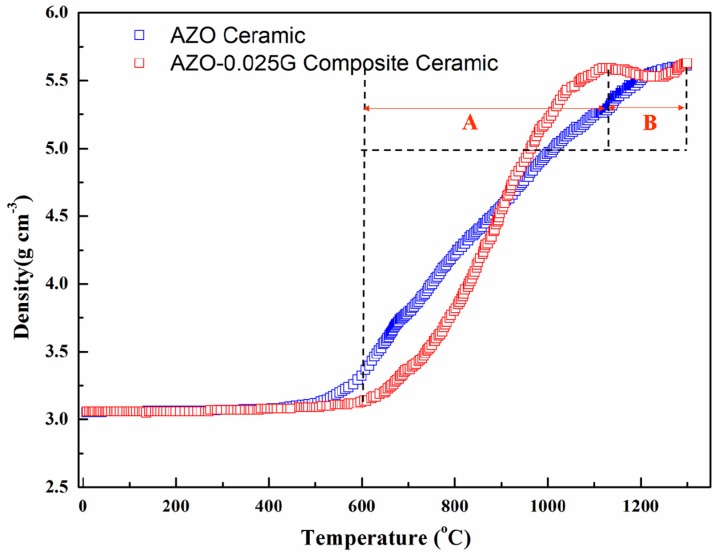
Normalized shrinkage of AZO ceramic and AZO-0.025G composite ceramics.

Table 1 shows that the addition of the appropriate amount of GNSs increases the relative density of AZO ceramics. However, the relative density of the AZO-0.025G composite ceramics exhibits a sharp increase as the sintering temperature increases to 1300 °C. To provide insight into the influence of GNSs on the sintering behavior of AZO ceramics, shrinkage as a function of the SPS sintering temperature for both the AZO ceramics and the AZO-0.025G composite ceramics is examined in the present study. Figure 7 shows the normalized densification behavior of the AZO and AZO-0.025G ceramics sintered at 100 °C/min and 40 MPa. The density values were calculated from the shrinkage curves by correcting for the thermal expansion of the graphite die, which was separately measured using dummy tests at the respective SPS conditions. As illustrated, the spark plasma sintering of AZO ceramics begins to shrink at ~400 °C and is complete at ~1200 °C, and hence follows a one-step densification. In contrast, the sintering behavior of AZO-0.025G shows a two-step shrinkage response. The first shrinkage occurs between ~600 and ~1100 °C (denoted as region A) and the second stage starts at ~1250 °C (denoted as region B). It is worth mentioning that the density of AZO-0.025G composite ceramics decreases in the sintering range from 1100 to 1200 °C, which is consistent with the porosity observed in AZO-0.025G at a sintering temperature of 1200 °C shown in Figure 3c. The underlying mechanism that results in the loss of GNSs at a higher sintering temperature is explained as follows. Graphene nanosheets can react with O_2_ to form CO or CO_2_, because Al doping is supposed to lead to an increase of the charge carrier concentration following Equation (1) [26]:

(1) $$Al_{2}O_{3}\overset{\;Z\text{nO}\;}{\to }2Al_{Zn}^{•}+2e^{'}+\frac{1}{2}O_{2}↑$$

When increasing the sintering temperature from 1200 to 1300 °C, graphene nanosheets are completely oxidized by oxygen from Equation (1). Thus, the porosity in AZO-0.025G composite ceramics decreases as the sintering temperature increases to 1300 °C. Although shrinkage of AZO ceramics occurs at a lower temperature of 400 °C, it is observed that fully dense AZO-0.025G composite ceramics are obtained at a lower temperature at 1100 °C, which is 100 °C lower than that required by AZO ceramics. With the addition of GNSs, significant increases in densification have been also observed for conductive ceramics such as TaC [25] and ZrB_2_ [27]. The higher conductivity enables significant current to flow directly through the sample during the SPS process and hence these ceramics benefit from both the increased thermal and electrical conductivity of graphene.

### 4.2. Influence of GNSs on Electrical Properties of AZO-G

The resistivity of AZO ceramics is primarily determined by the carrier concentration (*n*) and Hall mobility (*μ*), as shown by the following equation:

(2) $$\rho \equiv \frac{1}{n\mu e}$$

where *e* is a constant of electron charge (*e* = 1.602 × 10^−19^ C), n is the carrier concentration, *μ* is the Hall mobility. It is obvious that the resistivity of AZO ceramics is determined by the carrier concentration and Hall mobility, which can be seen from Equation (2). The carrier concentration (*n*) of AZO is determined by the substitution of Al^3+^ on Zn^2+^ and oxygen loss [28]. Hall mobility is influenced by impurity scattering (*μ*_i_), grain boundary scattering (*μ*_g_) and lattice thermal vibration scattering (*μ*_l_), as shown in the following expression [29,30]:

(3) $$\frac{1}{\mu }=\frac{1}{\mu_{i}}+\frac{1}{\mu_{g}}+\frac{1}{\mu_{l}}$$

The thermal vibration scattering effect is very weak at room temperature and can be neglected. Therefore, impurity scattering and grain boundary scattering are the main factors that limit Hall mobility. As the sintering temperature exceeds 1000 °C, Al cannot fully dissolve into the ZnO structure as the solubility of Al in ZnO is very small [28]. Thus, further Al additions leads to the formation of the non-conductive ZnAl_2_O_4_ phase and the ZnAl_2_O_4_ is segregated to grain boundaries and grain boundary triple junctions, which effectively decreases the electrical and thermal conductivities [26].

**Figure 8:**
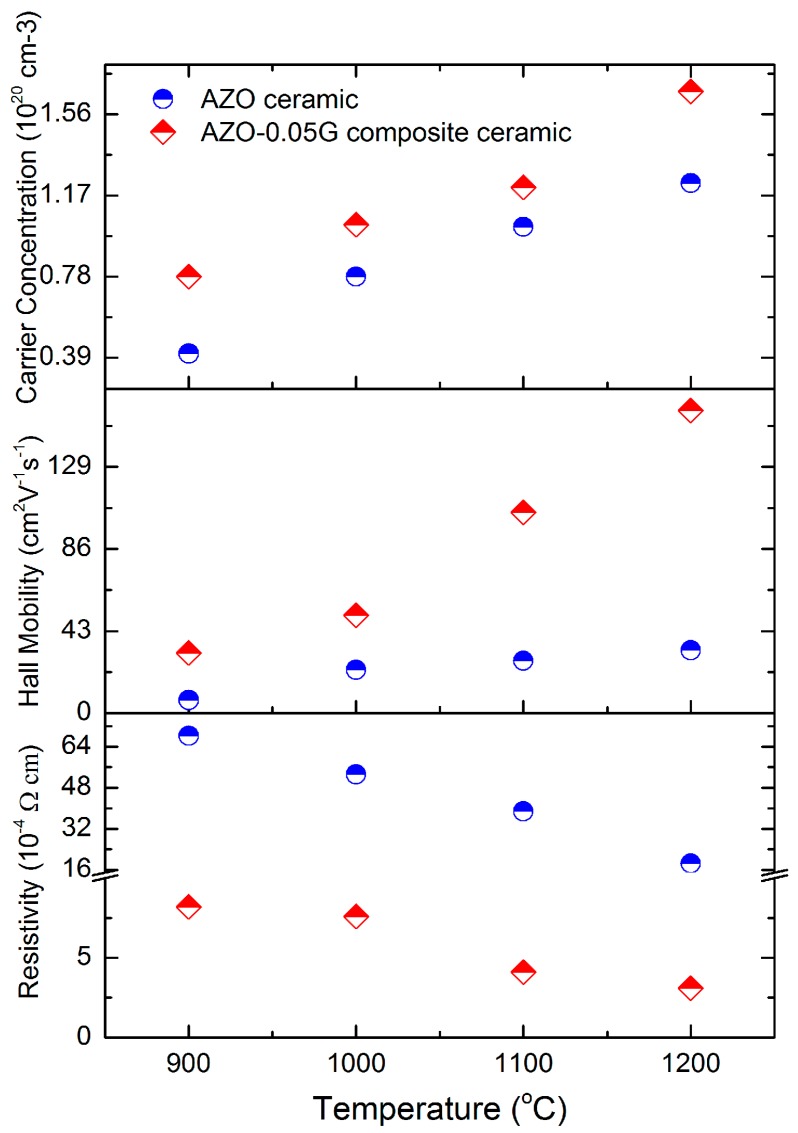
The resistivity, Hall mobility and carrier concentration for AZO ceramics and AZO-0.05G composite ceramics.

Figure 8 shows the relationship among resistivity, Hall mobility and carrier concentration for AZO and AZO-0.05G composite ceramics at various sintering temperatures. The AZO-0.05G composite ceramics show a fairly low resistivity of 3.1 × 10^−4^ Ω·cm, while the lowest resistivity of 1.7 × 10^−3^ Ω·cm for AZO ceramics is one order of magnitude than that of AZO-0.05G composite ceramics. Moreover, the addition of GNSs for AZO-0.05G composite ceramics significantly enhances the Hall mobility with a value from 27.4 to 105.1 cm^2^·V^−1^·s^−1^ which effectively enhances the resistivity.

In the case of AZO-G composite ceramics, the enhancement of electrical conductivity is principally attributable to the addition of GNSs in the AZO matrix. To that effect, there are two factors related to the enhancement of the conductivity of AZO-G composite ceramics: (1) the significantly higher electron mobility of GNSs relative to that of AZO; and (2) an efficient and integrated conductive network of GNSs in the copper matrix. In other words, when the volume fraction of GNSs is low, it is not possible to attain the continuous network needed to result in an overall increase in electrical conductivity. However, if the GNS content is too high, agglomeration can lead to microstructural flaws such as the formation of pores and cracks, which contribute to carrier scattering, thereby leading to an electrical conductivity decrease. Moreover, it is worth noting that the formation of the spinel-phase ZnAl_2_O_4_ contributes to a high degree of impurity scattering, both at grains and grain boundaries [31], which can be both significant and difficult to ameliorate [28]. Our work shows that by adding GNSs to an AZO matrix, ZnO/ZnO and ZnO/ZnAl_2_O_4_ grain boundaries play a role in the overall electrical response. For example, the grain boundary triple junction architecture shown in Figure 5 suggests that GNSs can provide efficient electron transport pathways in local regions including those associated with non-conductive ZnAl_2_O_4_ grains.

## 5. Conclusions

AZO-G composite ceramics with uniformly dispersed GNSs have been prepared via ball milling using dimethyl formamide followed by SPS. In the case of AZO-0.025G, a high relative density of 99.2% was attained at a sintering temperature of 1100 °C, which is comparable to AZO ceramics sintered at a sintering temperature of 1200 °C. The resistivity of AZO-0.05G composite ceramics sintered at 1200 °C shows a minimum value of 3.1 × 10^−4^ Ω·cm with a superior Hall mobility of 105.1 cm^2^·V^−1^·s^−1^. For pure AZO ceramics, the resistivity was 1.7 × 10^−3^ Ω·cm and the Hall mobility was 27.4 cm^2^·V^−1^·s^−1^. The improved electrical resistivity of the AZO-G composite ceramics is attributable to the distribution of GNSs at ZnO/ZnO grain boundaries, ZnO/ZnAl_2_O_4_ grain boundaries, and the grain boundary triple junctions. Moreover, the network of GNSs provides efficient electron transport pathways in local regions, including those associated with non-conductive ZnAl_2_O_4_ grains.

## Figures and Tables

**Table 1:** Relative density and average grain size of the sintered samples.

GNSs (wt. %)	Temperature (°C)	Relative Density (%)	Average Grain Size (μm)
0	1000	97.8	2.1
0	1100	98.4	3.3
0	1200	99.3	5.3
0.025	1000	98.3	1.8
0.025	1100	99.2	2.8
0.025	1200	97.2	4.3
0.05	1100	98.3	1.5
0.1	1100	97.2	0.9
